# Focusing on Good Responders to Pneumococcal Polysaccharide Vaccination in General Hospital Patients Suspected for Immunodeficiency. A Decision Tree Based on the 23-Valent Pneumococcal IgG Assay

**DOI:** 10.3389/fimmu.2019.02496

**Published:** 2019-11-05

**Authors:** Lisanne M. A. Janssen, Michiel Heron, Jean-Luc Murk, Alexander C. A. P. Leenders, Ger T. Rijkers, Esther de Vries

**Affiliations:** ^1^Department of Tranzo, Tilburg University, Tilburg, Netherlands; ^2^Department of Pediatrics, Amalia Children's Hospital, Nijmegen, Netherlands; ^3^Laboratory of Medical Microbiology and Immunology, St. Elisabeth Hospital Tilburg, Tilburg, Netherlands; ^4^Laboratory of Medical Microbiology, Jeroen Bosch Hospital, 's-Hertogenbosch, Netherlands; ^5^Science Department, University College Roosevelt, Middelburg, Netherlands

**Keywords:** primary immunodeficiency, humoral immunodeficiency, pneumococcal polysaccharide response, serotype-specific assay, polysaccharide response, pneumococcal vaccination response, 23-valent IgG assay, VaccZyme™

## Abstract

**Background and Aim:** Recently, the 23-valent IgG-assay was suggested as screening assay to identify *poor* responders to pneumococcal polysaccharide (PnPS)-vaccination with the serotype-specific assay as a second-line test. However, in a low pre-test probability general hospital setting predicting *good* responders could be more valuable to reduce the number of samples needing serotyping.

**Methods:** Serotype-specific PnPS antibody-assays were performed for suspected immunodeficiency in two Dutch general hospitals (Jeroen Bosch Hospital, 's-Hertogenbosch; Elisabeth Tweesteden Hospital, Tilburg). 23-Valent PnPS antibody-assays were subsequently performed in archived material. Data were analyzed using receiver operating characteristic curves (AUC) and agreement indices (ICC).

**Results:** Sera of 284 patients (348 samples) were included; 23-valent IgG-titres and the corresponding sum of PnPS-serotype specific antibodies showed moderate correlation (ICC = 0.63). In 232 conjugated-pneumococcal-vaccine-naïve patients (270 samples), a random 23-valent IgG-titer could discriminate between samples with and without ≥7/11, ≥7/13, or ≥6/9 pneumococcal serotypes when both cut-off values 0.35 and 1.0 μg/ml were used (AUC 0.86 and 0.92, respectively). All patients with a pre-immunization-titer ≥38.2 μg/ml and/or post-immunization-titer ≥96.1 μg/ml and none with a post-immunization-titer ≤38.5 μg/ml exhibited a good response to PnPS vaccination. Using these breakpoints as screening test to predict *good* responders, only 24% of patients would require further serotyping, as opposed to 68% if breakpoints to predict *poor* responders would have been used.

**Conclusion:** In a low pre-test probability setting, the 23-valent IgG-assay proved to be a reliable screening test for good responders in conjugated-pneumococcal-vaccine-naïve patients, reducing the overall number of patient samples needing further serotyping, thus reducing overall costs of pneumococcal vaccination response assessment.

## Introduction

Serotype-specific pneumococcal polysaccharide (PnPS) antibody testing is currently accepted as the “gold standard” (1–4) for the evaluation of anti-polysaccharide antibody production capacity in patients who are suspected to have primary antibody deficiency because of unexplained or recurrent (mainly respiratory) infections (4, 5). However, serotype-specific PnPS testing is not widely available and is time consuming, labor intensive and expensive. Moreover, uniform reference values are not available, and interpretation is therefore challenging (6–10).

Recent data has indicated that one-step measurement of the summated response to all 23 serotypes present in the polysaccharide pneumococcal vaccine (here called “23-valent IgG assay”) could be used as a screening test to reduce the overall number of patient samples needing serotyping (11, 12). This could significantly improve efficiency and reduce overall costs. In addition, this assay is widely available as in-house assay or easy-to-use commercial kit, and the test result is easy to interpret based on a single cut-off value (13). Given these advantages, the 23-valent IgG assay has been proposed to be used as a first-line test to identify clear-cut poor responders, and the serotype-specific assay as a second-line test for assessment of the PnPS vaccination response in non-clear-cut cases only. In their tertiary-center adult cohort (*n* = 62), Lopez et al. identified a cut-off value of 110 μg/ml, which was constantly associated with a poor response to PnPS vaccination using the serotype-specific assay (11).

However, *on a population basis*—i.e., in the context of a low pre-test probability setting—a screening method that can reliably predict *good* responders could be of greater value. After all, many patients with recurrent infections do not have an immunodeficiency. Or they suffer from milder forms of hypogammaglobulinemia, such as selective anti-polysaccharide antibody deficiency (SPAD) only (or combinations with IgG-subclass and/or IgA deficiency), without significantly decreased total immunoglobulin levels. These patients generally present themselves in secondary care, where the pre-test probability for severe antibody deficiency is inherently low. However, even milder hypogammaglobulinemia can lead to serious problems, requiring adequate medical attention (14). These milder patients are often not recognized due to lack of available test facilities in secondary care, and reluctance to refer many patients to an immunologist. Easy, reliable selection of patients can create support for a lower screening threshold for antibody deficiency in patients with recurrent infections in secondary care. Ultimately, this will help timely detection of all patients who do have an immunodeficiency. Our study was designed to investigate the suitability of the one-step summated response test for this purpose.

## Materials and Methods

### Study Design

Between February 2012 and December 2018, serotype-specific PnPS assays were performed on 348 blood samples in regular patient care, obtained from 284 patients who were analyzed for potential immunodeficiency in two secondary centers in the Netherlands [Jeroen Bosch Hospital, 's-Hertogenbosch (*n* = 234), Elisabeth Tweesteden Hospital, Tilburg (*n* = 50)]. Of these, 78 samples were from 64 patients who were previously vaccinated with conjugated pneumococcal vaccine (Pn-C). Left-over samples were stored at ≤-80°C and later retrieved from the laboratory to perform 23-valent pneumococcal IgG assays. The research project was granted ethical approval by the local medical ethics committee and consent was obtained from all adults and parents of the children.

### Test Methods

#### The Clinical Reference Standard

The IgG antibodies against PnPS were measured on a Luminex platform using a quantitative multiplex immunoassay including cell wall polysaccharide (CPS) and 22F adsorption (15). For the Jeroen Bosch Hospital, this serotype-specific assay was performed in the Department of Medical Immunology, University Medical Center Utrecht, the Netherlands. Titers were assessed against eleven serotypes (1, 3, 4, 5, 6B, 7F, 9V, 14, 18C, 19F, 23F) until February 2014, and thereafter against nine serotypes (6B, 8, 9V, 14, 15B, 19F, 20, 23F, 33F). For the Elisabeth Tweesteden Hospital, this assay was performed in the St. Antonius Hospital, Nieuwegein, the Netherlands. In this laboratory, titers were assessed against thirteen serotypes (1, 3, 4, 5, 6A, 6B, 7F, 9V, 14, 18C, 19A, 19F, 23F); in a subset of these samples (*n* = 132), 22 of the serotypes present in the 23-valent IgG assay (all except 17F) were determined. For the interpretation of PnPS serotype concentrations two different thresholds were used: ≥0.35 and ≥1.0 μg/ml (based on protection against invasive infection and colonization, respectively) (10, 16–19). For both limits, sufficient levels were defined in vaccine-naïve patients as ≥7/11, ≥7/13, or ≥6/9 serotypes reaching these concentrations (based on the reference values of the respective laboratories). In 174/284 (61%) patients a blood sample was drawn 4–8 weeks after intramuscular vaccination with one dose of 23-valent PnPS vaccine (Pneumovax 23; Merck, Sharp & Dohme BV, Haarlem, The Netherlands) containing 23 μg purified type-specific capsular polysaccharide of 23 pneumococcal serotypes (1, 2, 3, 4, 5, 6B, 7F, 8, 9N, 9V, 10A, 11A, 12F, 14, 15B, 17F, 18C, 19F, 19A, 20, 22F, 23F, and 33F; Danish nomenclature). A positive response to PnPS vaccination was defined according to the guidelines of the laboratory (20, 21). Briefly, a good response to PnPS vaccination was defined by a post-immunization titer ≥1.0 μg/ml in ≥7/11, ≥7/13, or ≥6/9 serotypes in patients not previously immunized with Pn-C vaccine. In Pn-C pre-vaccinated patients, the serotypes not present in the vaccine were evaluated (Utrecht; when vaccinated with Pn-C7, serotypes 1, 3, 5, 7F; when vaccinated with Pn-C10, serotypes 8, 15B, 20, 33F|Nieuwegein; when vaccinated with Pn-C7, serotypes 1, 3, 5, 6A, 7F, 19A; when vaccinated with Pn-C10, serotypes 3, 6A, 19A). According to the Utrecht laboratory's reference values, we corrected for age in these samples: for ages 4–6 years, an abnormal result was defined as <50% of serotypes evaluated reaching an IgG titer of ≥1 μg/ml. For age ≥6 years, an abnormal result was defined as <75% of serotypes evaluated reaching an IgG titer of ≥1 μg/ml. According to the Nieuwegein laboratory's reference values, an abnormal result was defined as <70% of serotypes evaluated reaching an IgG titer of ≥1 μg/ml in those samples.

#### The Index Test

For the measurement of the 23-valent IgG titer, the VaccZyme™ anti-PCP IgG ELISA Kit (The Binding Site, Birmingham, United Kingdom) with precoated microtiter plates was used according to the manufacturer's instructions (22, 23). Absorption of interfering anti-cell wall polysaccharide (CPS) antibodies was incorporated in this assay. The VaccZyme™ anti-PCP IgG assay was performed in the Laboratory of Medical Microbiology and Immunology at the Elisabeth Tweesteden Hospital (Tilburg, the Netherlands).

### Statistical Analysis

Data were analyzed using SPSS 24.0 software for Mac. Differences in measurements were tested with *t*-test (Welch's *t*-test when the variances are unequal) and ANOVA. Separate analyses were performed for patients previously immunized with Pn-C. Correlation between the 23-valent IgG titer and the sum of the serotype-specific antibody titers in the same sample was assessed with the intraclass correlation coefficient (ICC). The strength of the relationship for the ICC coefficient r was classified as follows: 0.3 ≤ *r* < 0.5 “poor,” 0.5 ≤ *r* < 0.75 “moderate,” 0.75 ≤ *r* < 0.9 “good” and 0.9 ≤ *r* < 1.0 “excellent” (24). To determine whether a random 23-valent IgG titer could predict that ≥7/11, ≥7/13, or ≥6/9 (vaccine-naïve patients) vs. ≥2–3/4, ≥2/3, or ≥4/6 (Pn-C pre-vaccinated patients) serotypes were above the two different cut-off levels (≥0.35 or ≥1.0 μg/ml) in the same sample, receiver operating characteristics (ROC) curves were plotted and the area under the curves (AUCs) were calculated. To determine whether the pre-, or 4–8 weeks post-immunization 23-valent IgG titer could predict if that patient would become a good or poor responder to PnPS vaccination as assessed by the serotype-specific assay, also for these variables ROC curves were plotted and AUCs calculated. The best cut-off values were chosen according to (1) the Youden index calculation, (2) the maximum sensitivity for pre-immunization 23-valent IgG titers, and (3) the maximum sensitivity and specificity for 4–8 weeks post-immunization 23-valent IgG titers. For each identified cut-off value, the positive and negative predictive values were calculated. All tests were two-tailed and *p*-values <0.05 were considered to be statistically significant.

## Results

### Participants

127/284 (45%) patients were females, and the mean age at inclusion was 36.3 years (range 1.1–89.7). In 54 patients, two or more different samples were available, resulting in 348 samples with paired serotype-specific and 23-valent pneumococcal IgG titers available for analysis [78/348 (22%) samples from patients previously immunized with Pn-C vaccine]. Of the 270 samples from patients not previously immunized with Pn-C vaccine, 194 were pre-immunization samples, 38 were 4–8 weeks post-immunization samples, and 38 were >8 weeks post-immunization samples (mean duration after vaccination 33 months, range 15–70 months). Of the 78 samples from patients previously immunized with Pn-C vaccine, 63 were pre-immunization samples, 14 were 4–8 weeks post-immunization samples, and 1 was a >8 weeks post-immunization sample (28 months).

### Test Results

**Figure 1:**
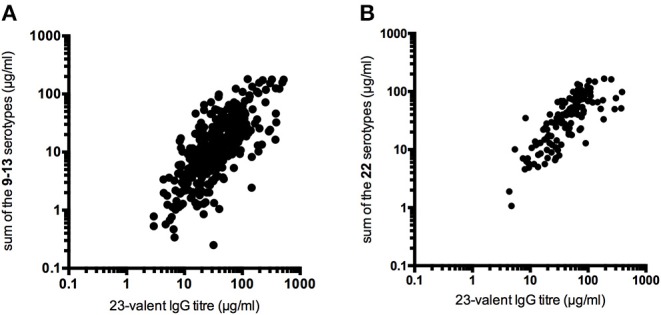
Correlation between the 23-valent pneumococcal IgG titer and the sum of the pneumococcal polysaccharide serotype titers determined in the same sample (**A**: sum of 9–13 individual serotypes, 348 samples, ICC = 0.65, 95% CI = 0.57–0.72, *P* < 0.0001; **B**: sum of 22 individual serotypes, 132 samples, ICC = 0.64, 95% CI = 0.49–0.75).

In all samples taken together, a moderate correlation between the sum of the individual PnPS serotypes and the 23-valent IgG titer in the same sample was observed (ICC = 0.65, 95% CI = 0.57–0.72, *P* < 0.0001; Figure 1A). The ICC did not improve when the sum of a larger set of 22 individual PnPS serotypes was plotted against the 23-valent IgG titer (available for 132 samples; ICC = 0.64, 95% CI = 0.49–0.75; Figure 1B).

#### Patients Not Previously Immunized With Conjugated Pneumococcal Vaccine

**Figure 2:**
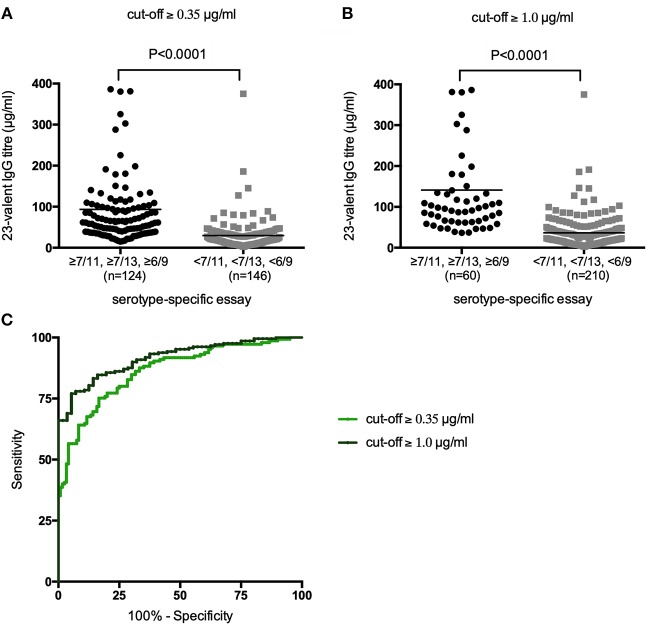
23-Valent pneumococcal IgG titers (μg/ml) in samples with and samples without ≥7/11, ≥7/13, or ≥6/9 serotypes above the cut-off values of 0.35 μg/ml **(A)** and 1.0 μg/ml **(B)**. *P*-values were calculated in an unpaired *t*-test. Receiver operating characteristic (ROC) curves of sensitivity vs. specificity of the 23-valent pneumococcal IgG titer using two different cut-off levels for the serotype-specific assay **(C)**.

First, all samples from patients not previously immunized with conjugated pneumococcal vaccine were analyzed together, irrespective of whether the patients had received PnPS-vaccination. The 23-valent IgG titers were significantly higher in samples with ≥7/11, ≥7/13, or ≥6/9 serotypes above both the cut-off levels 0.35 and 1.0 μg/ml, respectively, compared to samples with <7/11, <7/13, or <6/9 serotypes above these cut-off levels (*p* < 0.0001 for both cut-off levels; Figure 2). A 23-valent IgG titer could discriminate between samples with and without ≥7/11, ≥7/13, or ≥6/9 serotypes above both the cut-off values 0.35 and 1.0 μg/ml [ROC analysis; AUC 0.86 (95% CI 0.82–0.91) and 0.92 (95% CI 0.88–0.95), respectively; Figure 2, Table 1]. Based on the calculation of the Youden index, the best threshold was a 23-valent IgG titer of ≤38.2 μg/ml for the serotype-specific cut-off level of 0.35 and ≤54.2 μg/ml for the cut-off level 1.0 μg/ml. However, neither of them achieved estimates of both sensitivity and specificity >86% (Table 1).

**Table 1:** 23-Valent pneumococcal IgG titers compared to the serotype-specific assay in the same sample (*n* = 270).

**A. Estimated areas under the curve (AUCs) with their (95% CI) and** ***p*****-values**						
	**AUC (95% CI)**	***P*****-value**	**Youden index**	**Best-choice criterion according to:**		
				**Youden index calculation**	**100% Se**	**100% Sp**
Cut-off ≥0.35 μg/ml	0.86 (0.82–0.91)	<0.0001	0.55	≤38.2 μg/ml	≥188.5 μg/ml	≤16.1 μg/ml
Cut-off ≥1.0 μg/ml	0.92 (0.88–0.95)	<0.0001	0.69	≤54.2 μg/ml	≥188.5 μg/ml	≤36.8 μg/ml
**B. Performance indicators and levels of agreement for the 23-valent pneumococcal IgG titers**						
**Criterion**	**Se (%)**	**Sp (%)**	**PPV (%)**	**NPV (%)**	**McNemar's test** ***p*****-value**	**Cohen's kappa**
**Cut-off value 0.35** **μg/ml**						
≤38.2^a^	79.3 (71.8–85.6)	75.8 (67.2–83.2)	79.3 (73.5–84.1)	75.2 (68.6–80.8)	1.000	0.55
**Cut-off value 1.0** **μg/ml**						
≤54.2^a^	83.3 (77.5–88.1)	85.7 (73.8–93.6)	95.6 (91.9–97.7)	59.1 (51.4–66.4)	<0.0001	0.60

a Selected using the Youden index. All statistics are presented with the corresponding (95% CI).

*CI, confidence interval; IgG, immunoglobulin G; NPV, negative predictive value; PPV, positive predictive value; Se, sensitivity; Sp, specificity*.

**Figure 3:**
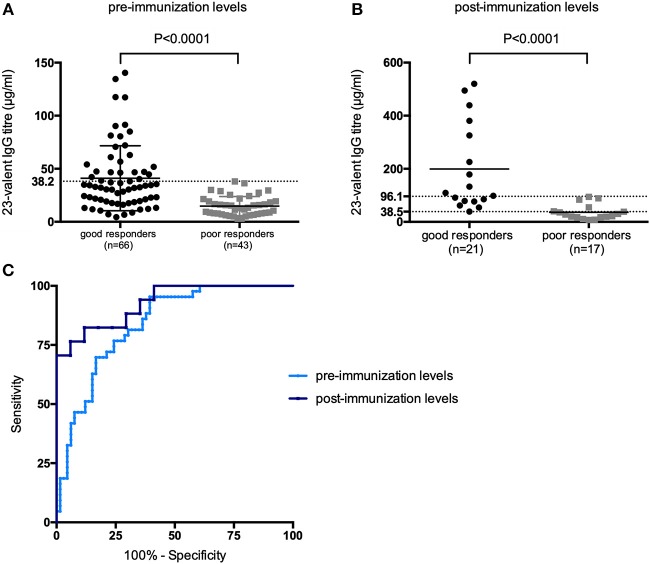
23-Valent pneumococcal IgG titers (μg/ml) for good vs. poor responders, using the two indicated parameters in a per patient analysis: **(A)** 23-valent pre-immunization IgG levels, **(B)** 23-valent post-immunization IgG levels in good- and poor responders to pneumococcal polysaccharide vaccination. *P*-values were calculated in an unpaired *t*-test. Receiver operating characteristic (ROC) curves of sensitivity vs. specificity for the 23-valent pre- and 4–8 weeks post-immunization levels vs. serotype-specific response to vaccination **(C)**.

**Figure 4:**
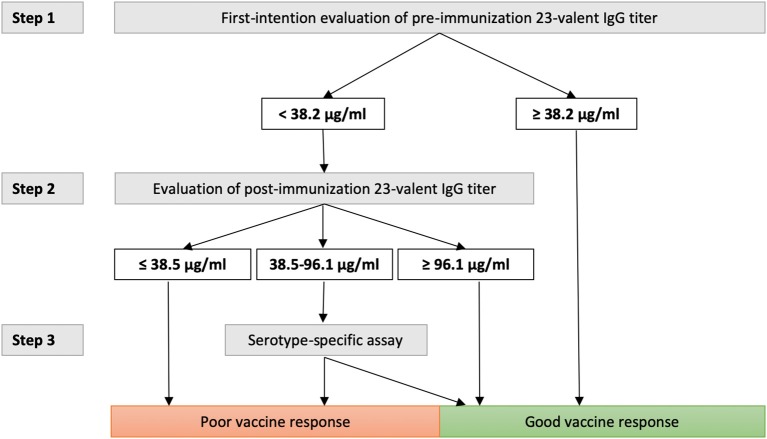
Decision tree using the 23-valent pre-immunization titer as a first-line test, 23-valent 4–8 weeks post-immunization titer as second-line test, and serotype-specific assay for definitive assessment of response, if indicated. The cut-off levels were determined only on patients who were not previously immunized with conjugated pneumococcal vaccine (see Results section and Figure 3).

Next, the pre- and 4–8 weeks post-immunization 23-valent IgG measurements were compared with the serotype-specific PnPS vaccination response to perform a *per patient* analysis (information available for 147 patients). This is clinically the most interesting evaluation. Sixty (41%) patients were defined as poor responders according to the serotype-specific assay. For both 23-valent pre- and 4–8 weeks post-immunization titers a significant difference was observed between good and poor responders (*p* < 0.0001; Figure 3). The results of ROC curve analyses of the 23-valent IgG assay performance vs. the serotype-specific assay per patient are shown in Figure 3 and Table 2. Because Cohen's kappa was never better than moderate (<0.75), this ruled out the use of the 23-valent IgG assay alone and implicated the necessity of a stepwise approach. We wanted to establish whether use of the 23-valent IgG assay alone could reliably discriminate good responders, who do not need any further diagnostic work-up, as well as poor responders, whom we do not want to miss. We therefore favored a high sensitivity for *pre*-immunization titers, and both high specificity as well as high sensitivity for post-immunization titers. All patients with a 23-valent pre-immunization titer ≥38.2 μg/ml and/or post-immunization titer ≥96.1 μg/ml and none of the patients with a post-immunization titer ≤ 38.5 μg/ml exhibited a good response to PnPS vaccination (Table 2B). Based on these data, a stepwise approach was developed using both tests (Figure 4). 24% (26/109) of patients had a pre-immunization 23-valent IgG level ≥38.2 μg/ml, 32% (12/38) of patients had a post-immunization 23-valent IgG level ≤38.5 μg/ml; 37% (14/38) of patients had a post-immunization 23-valent IgG level ≥96.1 μg/ml (in four of these patients also a pre-immunization sample was available). A scenario using the 23-valent IgG assay as pre-screen would have decreased costs in our general hospital patient population from $46.00 down to $20.37 per patient (based on global average test prices of the 23-valent IgG assay of $5.30 per well, and of the serotype-specific assay of $46.00 per sample, and on the assumption that all wells are used in the test run; calculation: (100 × 5.30 + 76 × 5.30 + 24 × 46.00)/100 = 20.37) (25).

**Table 2:** 23-Valent pneumococcal IgG titers compared to serotype-specific response to pneumococcal vaccination in patients not previously immunized with conjugated pneumococcal vaccine.

**A. Estimated areas under the curve (AUCs) with their (95% CI) and** ***p*****-values**						
	**AUC (95%CI)**	***P*****-value**	**Youden index**	**Best-choice criterion according to:**		
				**Youden index calculation**	**100% Se**	**100% Sp**
Pre-immunization levels (*n* = 109)	0.84 (0.76–0.91)	<0.0001	0.51	≤22.4 μg/ml	≥38.2 μg/ml	NC
4–8 weeks post-immunization levels (*n* = 38)	0.93 (0.85–1.00)	<0.0001	0.71	≤ 58.3 μg/ml	≥96.1 μg/ml	≤38.5 μg/ml
**B. Performance indicators and levels of agreement for the 23-valent IgG titers**						
**Criterion**	**Se (%)**	**Sp (%)**	**PPV (%)**	**NPV (%)**	**McNemar's test** ***p*****-value**	**Cohen's kappa**
**Pre-immunization titer (μg/ml)**						
≤22.4^a^	81.4 (66.6–91.6)	69.7 (57.2–80.4)	63.6 (54.2–72.2)	85.2 (75.1–91.6)	0.003	0.44
**≥38.2**	**100.0 (91.8–100.0)**	**39.9 (27.6–52.2)**	**48.2 (43.6–52.8)**	**100.0**	** <0.0001**	**0.34**
**4–8 weeks post-immunization titer (μg/ml)**						
≤58.3^a^	82.4 (56.6–96.2)	88.2 (63.6–98.5)	87.5 (64.8–96.4)	86.4 (69.2–94.7)	1.000	0.73
**≤38.5**	**70.6 (44.0–89.7)**	**100.0 (80.5–100.0)**	**100.0**	**80.8 (66.8–89.8)**	**0.063**	**0.73**
≥**96.1**	**100.0 (80.5–100.0)**	**58.8 (32.9–81.6)**	**66.7 (53.5–77.7)**	**100.0**	**0.016**	**0.64**

a Selected using the Youden index. The selected threshold's performance is highlighted in bold font. All statistics are presented with the corresponding (95% CI).

*CI, confidence interval; IgG, immunoglobulin G; NC, not calculated; NPV, negative predictive value; PPV, positive predictive value; Se, sensitivity; Sp, specificity*.

**Figure 5:**
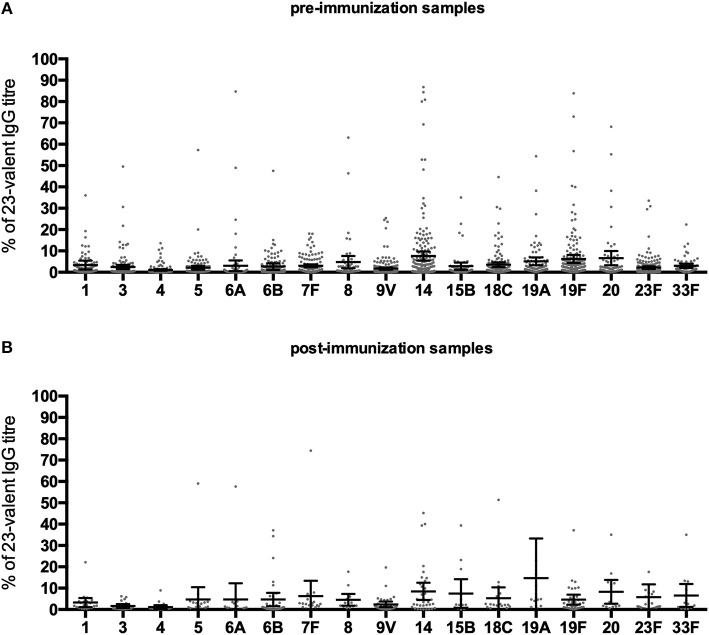
Percentage that a serotype contributed to the 23-valent IgG titer in pre-immunization samples **(A)**, and post-immunization samples **(B)** in Pn-C vaccine-naïve patients. Error bars represent mean percentages +95% CI.

Antibody levels against individual serotypes differed considerably, with serotypes 14 and 19F being dominant in most cases (Figure 5). On average, antibodies to individual serotypes contributed to the total 23-valent IgG titer ranging from 1.1 to 7.6% in pre-immunization samples, and 1.1 to 14.7% in post-immunization samples. In pre-immunization samples, serotype 14 contributed significantly more to the 23-valent IgG titer compared to serotypes 1, 3, 4, 5, 6A, 6B, 7, 9, 18C, and 23F (analysis of variance; *F* = 5.974, *p* = < 0.0001). In post-immunization samples, no overall dominant serotype could be identified (analysis of variance; *F* = 1.291, *p* = 0.20). In 19 pre-immunization and 4 post-immunization samples, antibodies against a single serotype contributed more than 50% to the 23-valent IgG titer, but only two of those pre-immunization samples and none of those post-immunization samples had a high 23-valent IgG titer (defined as ≥38.2 μg/ml pre-immunization and ≥96.1 μg/ml post-immunization, Supplementary Figure 1). In these two samples, the pre-immunization sample where serotype 19F dominated (73% of the 23-valent IgG titer), had 4/13 serotypes with a concentration ≥0.35 μg/ml and 2/13 serotypes ≥1.0 μg/ml. A post-immunization sample was not available for this patient, therefore it was not possible to classify this patient as good or bad responder to PnPS vaccination. In the pre-immunization sample where serotype 14 was dominant (84% of the 23-valent IgG titer), an additional 7/9 serotypes had concentrations above both cut-off levels 0.35 and 1.0 μg/ml (this patient can be considered a good responder based on natural exposure).

#### Patients Previously Immunized With Conjugated Pneumococcal Vaccine

First, all samples from patients who were previously immunized with conjugated pneumococcal vaccine were analyzed together (*n* = 78), irrespective of whether the patients had received PnPS-vaccination. The 23-valent IgG titers were higher in samples with ≥2–3/4, ≥2/3, or ≥4/6 serotypes above both the cut-off levels of 0.35 and 1.0 μg/ml, respectively, compared to the samples with <2–3/4, <2/3, or <4/6 serotypes above these cut-off levels (*p* = 0.013 for the cut-off level of 0.35 μg/ml, and *p* = 0.058 for the cut-off level of 1.0 μg/ml; Supplementary Figure 2). The 23-valent IgG titer could only fairly discriminate between samples with and without ≥2–3/4, ≥2/3, or ≥4/6 serotypes above both the cut-off values 0.35 and 1.0 μg/ml [ROC analysis; AUC 0.77 (95% CI 0.66–0.88) and 0.80 (95% CI 0.67–0.92), respectively; Supplementary Figure 1]. Based on the calculation of the Youden index, the best threshold was a 23-valent IgG titer of ≤50.2 μg/ml for the serotype-specific cut-off level of 0.35 μg/ml (sensitivity 76%, specificity 71%) and ≤58.4 μg/ml for the cut-off level 1.0 μg/ml (sensitivity 77%, specificity 83%).

Next, the pre- and 4–8 weeks post-immunization 23-valent IgG measurements were compared with the serotype-specific PnPS vaccination response to perform a *per patient* analysis (information available for 27 patients; 33% (26/78) of patients were <4 years and therefore not yet tested with PnPS vaccination). Eight (30%) patients were defined as poor responders according to the serotype-specific assay. For both 23-valent pre- and 4–8 weeks post-immunization titers, there was no significant difference between good and poor responders (pre-immunization titers: mean 43.8 μg/ml vs. 43.0 μg/ml, *p* = 0.584; post-immunization titers: mean 157.2 μg/ml vs. 148.0 μg/ml, *p* = 0.401). Because too few patients in our cohort were vaccinated with the 23-valent PnPS vaccine, test performance statistics could not be performed on these data.

## Discussion

We showed that a 23-valent IgG assay can be a reliable screening test to predict *good* responders to PnPS-vaccination in conjugated-vaccine-naïve patients in the low pre-test probability setting of a general hospital using our decision tree (Figure 4). The 23-valent IgG assay is widely available and easy to interpret. Implementing this procedure in general hospital care could lower the threshold for timely detection of primary antibody deficiency (PAD) by reducing the overall number of patient samples needing serotype specific antibody measurement, thus reducing overall costs. It is important to realize that recent studies focusing on using this assay as a first-line test were used to screen for *poor* responders to PnPS vaccination in highly selected patient populations referred to tertiary immunodeficiency expert centers (11, 12). Both approaches are valuable, but each should be used in the appropriate setting only.

We found that a cut-off value of ≥38.2 μg/ml in the pre-PnPS-immunization 23-valent IgG assay could reliably predict good responders in our cohort of conjugated-vaccine-naïve patients. This cut-off value is higher than the lower limit of the normal range (10.0, 11.0, and 15.4 μg/ml), and just below the means (41.0, 45.8, and 59.5 μg/ml) found in three previous studies of healthy vaccine-naïve adults using the same assay as our study (13, 26, 27). A post-PnPS-immunization threshold of ≤38.5 μg/ml yielded 100% specificity in conjugated-vaccine-naïve patients. This cut-off value is below the lower limit of the normal range post-vaccination (50 and 77 μg/ml) found in 2 studies of healthy unvaccinated adults using the same assay as our study (28, 29), and similar to the cut-off value ( ≤40 μg/ml) used by the Utrecht group (12, 22). It is also lower than found in a previous study by Lopez et al., were a 23-valent IgG titer of ≤110 μg/ml yielded a specificity of 100% and predicted 57% of the poor responders to PnPS vaccination (11). However, our data are not easily comparable to those of Lopez et al., as different sets and numbers of serotypes were tested in the serotype-specific assay, the criteria to define a deficient response were different, and most important, they investigated a highly selected patient population of which 75% was diagnosed with a humoral immunodeficiency. In contrast, good responders predominated in our general population cohort. This again emphasizes the importance of fitting the screening approach to the appropriate setting.

The performance of the 23-valent IgG assay was better in post-immunization 23-valent IgG sera (AUC 0.93) than in pre-immunization sera (AUC 0.84), which is consistent with previous studies (11, 27, 30).

In Pn-C pre-vaccinated patients, both pre- and post-23-valent IgG titers were similar in good and poor responders. This is in agreement with previous studies, in which it has been shown that the 23-valent IgG assay could not discriminate between good and poor responders to PnPS-vaccination in Pn-C pre-vaccinated patients (12, 31). Since a majority of childhood vaccination programs now include Pn-C vaccination, and a 20-valent conjugated vaccine is currently investigated with the intent to broaden global protection against pneumococcal disease, the future value of the 23-valent IgG assay as screening method for SPAD will probably become limited. Recent results show promising results for the measurement of the Typhim Vi IgG response as a diagnostic tool for assessing polysaccharide production in Pn-C pre-vaccinated patients (32).

The WHO recommended assay for measuring serotype-specific PnPS antibodies is by ELISA. A growing number of clinical laboratories, including ours, now are using multiplex bead technology for this purpose. The correlation between the two types of assays is good, although there can be variation in the absolute concentrations measured. It has been evaluated whether this variation would affect response classification of patients when using paired clinical sera (33). It was concluded that despite variation in absolute values of pneumococcal antibodies, the overall classification of the pneumococcal immune status of the patient was remarkably similar between assays (33, 34). In a recent publication it has been suggested to adjust multiplex cut-off values of selected polysaccharides to improve agreement level with WHO ELISA (35).

Our study has several limitations. First, a high 23-valent IgG titer corresponding with a good PnPS vaccination response by the serotype-specific assay may nevertheless not be protective because the antibody has low avidity or low opsonophagocytic activity. However, there are currently no accepted clinical criteria regarding normal PnPS-vaccination response based on measurements other than serotype-specific PnPS concentration (3). For future studies, it would be interesting to compare the 23-valent IgG assay with functional opsonophagocytosis assays. Second, the modest correlation between the 23-valent IgG titer and the sum of individual PnPS serotypes may be explained by the use of serotype-specific results expressed as “higher than” (for example >10 μg/ml). Determination of the exact concentration by titration of the sera, which has not been performed in this study, may result in a better agreement between both assays. It has long been recognized that the 23-valent IgG assay is of limited value in patients where isolated, elevated serotype titers are responsible for a high 23-valent IgG titer. While this may be true for selected patient cohorts, in a general patient population, such as ours, this turns out not to be the case. In only few samples (19/270 pre-immunization and 4/38 post-immunization), an isolated serotype contributed more than 50% to the 23-valent IgG titer, and a high 23-valent IgG titer was never present in the four post-immunization samples and only in 2/19 pre-immunization samples. Unfortunately, these two patients were not vaccinated, so it cannot be excluded that they could be incorrectly classified as “expected to be a good responder” by our decision tree. Last, it should be pointed out that in our per patient analysis, post-immunization samples obtained >8 weeks after vaccination were excluded. The number of those samples was too low to perform a meaningful comparison.

In conclusion, this is the first study evaluating the application of the 23-valent IgG VaccZyme™ anti-PCP IgG ELISA Kit for predicting *good* responders to PnPS-vaccination in a general hospital patient population setting. We showed that this assay can be a first screening test in Pn-C vaccine-naïve patients to determine which patients in a general hospital setting do *not* need serotype-specific testing. This can reduce the number of PnC vaccine-naive patients needing PnPS serotyping in the low pre-test probability setting of a general hospital, thus lowering the threshold for testing for suspected PAD while simultaneously reducing overall costs.

## Data Availability Statement

The datasets generated for this study are available on request to the corresponding author.

## Ethics Statement

The studies involving human participants were reviewed and approved by METC Brabant. Written informed consent to participate in this study was provided by the participants or their legal guardian/next of kin.

## Author Contributions

LJ, MH, and EV designed the study and wrote the manuscript. MH and EV acquired the data. LJ carried out the statistical analyses. GR helped with the interpretation of the data. J-LM, AL, and GR reviewed the results and contributed to the final version of the manuscript.

### Conflict of Interest

VaccZyme™ Anti-PCP human IgG ELISA kits were donated by the Binding Site Group Ltd., Birmingham, UK. Binding Site Group Ltd. had no role in the experimental design nor the analysis and interpretation of the data. The authors declare that the research was conducted in the absence of any commercial or financial relationships that could be construed as a potential conflict of interest.

## Supplementary Material

Supplementary Figure 1 Titers of serotypes that contributed ≥50% to the 23-valent IgG titer plotted against the 23-valent IgG titer in the same sample. Lines are drawn at the cut-off values as previously calculated (38.2 μg/ml for pre-immunization samples; 38.5 and 96.1 μg/ml for post-immunization samples).

Supplementary Figure 2 23-Valent pneumococcal IgG titers (μg/ml) in samples with and samples without ≥2–3/4, ≥2/3, or ≥4/6 serotypes above the cut-off values of 0.35 μg/ml **(A)** and 1.0 μg/ml **(B)**. *P*-values were calculated in an unpaired *t*-test. Receiver operating characteristic (ROC) curves of sensitivity vs. specificity of the 23-valent pneumococcal IgG titer using two different cut-off levels for the serotype-specific assay **(C)**.

## Abbreviations

AUC: area under the curve
CPS: capsular polysaccharide
ICC: intraclass correlation coefficient
PID: primary immunodeficiency
Pn-C: conjugated pneumococcal vaccine
PnPS: pneumococcal polysaccharide
ROC: receiver operating characteristics
SPAD: selective anti-polysaccharide antibody deficiency.
