# Supplementary figures and images for: Focusing on Good Responders to Pneumococcal Polysaccharide Vaccination in General Hospital Patients Suspected for Immunodeficiency. A Decision Tree Based on the 23-Valent Pneumococcal IgG Assay
Source: Front Immunol. 2019 Nov 5;10:2496. doi: 10.3389/fimmu.2019.02496 (PMC6848064; doi:10.3389/fimmu.2019.02496)

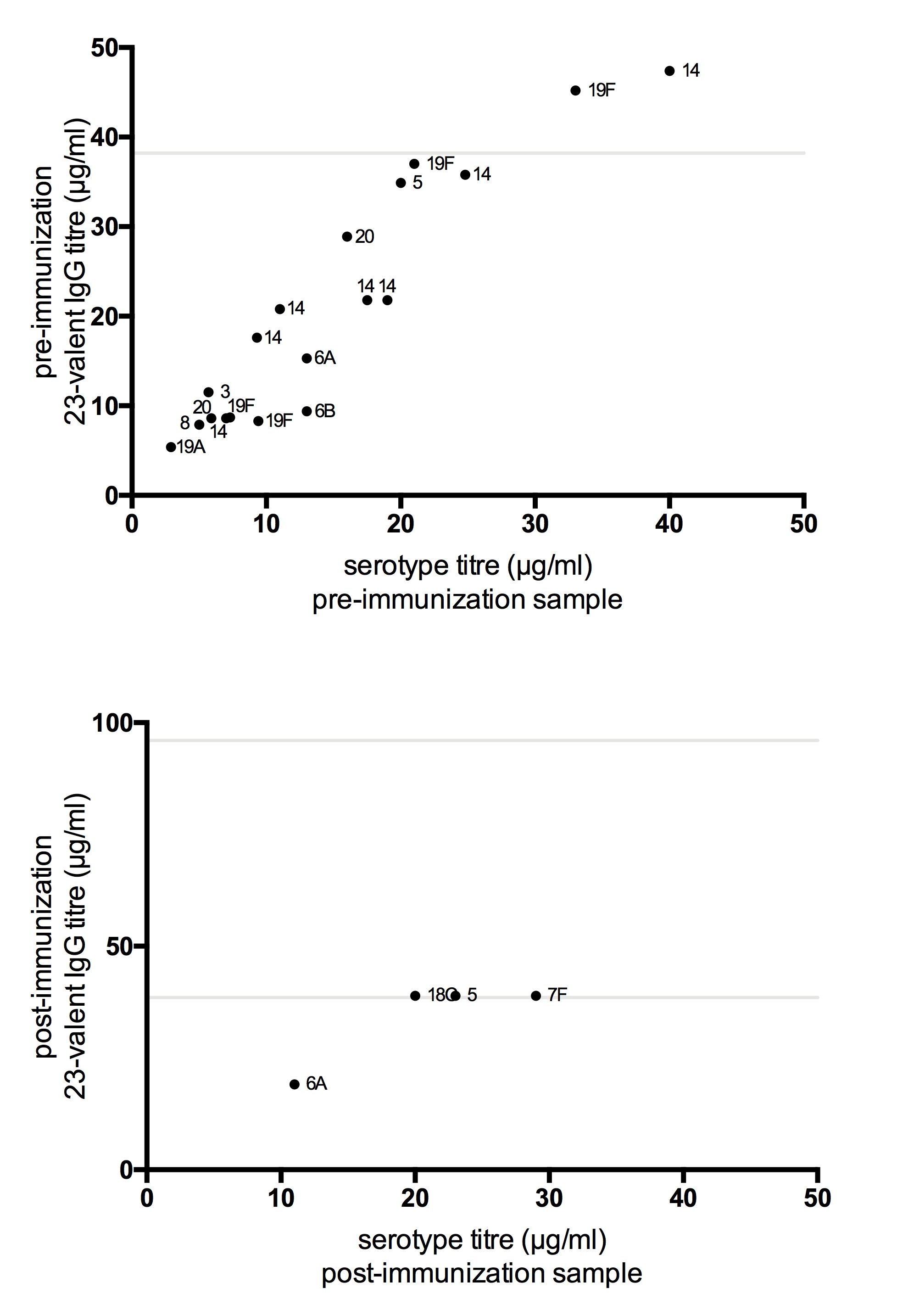

Supplement: Supplementary Figure 1 — Titers of serotypes that contributed ≥50% to the 23-valent IgG titer plotted against the 23-valent IgG titer in the same sample. Lines are drawn at the cut-off values as previously calculated (38.2 μg/ml for pre-immunization samples; 38.5 and 96.1 μg/ml for post-immunization samples). [file Image_1.TIFF]

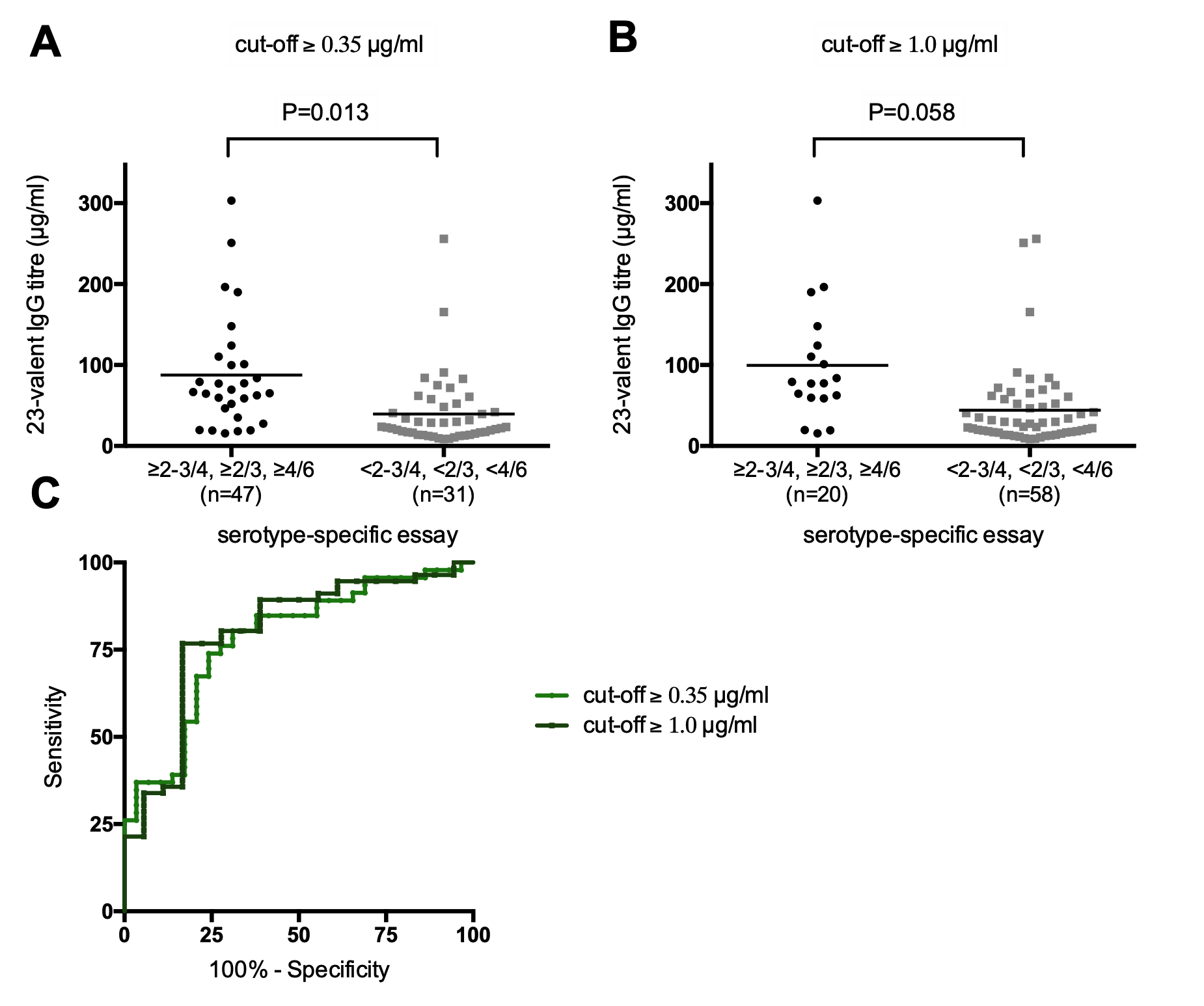

Supplement: Supplementary Figure 2 — 23-Valent pneumococcal IgG titers (μg/ml) in samples with and samples without ≥2–3/4, ≥2/3, or ≥4/6 serotypes above the cut-off values of 0.35 μg/ml (A) and 1.0 μg/ml (B). P-values were calculated in an unpaired t-test. Receiver operating characteristic (ROC) curves of sensitivity vs. specificity of the 23-valent pneumococcal IgG titer using two different cut-off levels for the serotype-specific assay (C). [file Image_2.tiff]
